# Comprehensive Safety Assessment of *Lacticaseibacillus paracasei* subsp. *paracasei* NTU 101 Through Integrated Genotypic and Phenotypic Analysis

**DOI:** 10.3390/cimb46110734

**Published:** 2024-11-01

**Authors:** Chieh-Ting Chen, Wen-Yu Chao, Chih-Hui Lin, Tsung-Wei Shih, Tzu-Ming Pan

**Affiliations:** 1 SunWay Biotech Co., Ltd., Taipei 114067, Taiwan; ct.chen@sunway.cc (C.-T.C.); wendy.chao@sunway.cc (W.-Y.C.); tw.shih@sunway.cc (T.-W.S.); 2 Department of Life Science, National Taitung University, Taitung 950309, Taiwan; chlin@nttu.edu.tw; 3 Department of Food Science, College of Human Ecology, Fu Jen Catholic University, New Taipei City 242062, Taiwan; 4 Department of Biochemical Science and Technology, National Taiwan University, Taipei 106319, Taiwan

**Keywords:** *Lacticaseibacillus paracasei* subsp. *paracasei* NTU 101, genotypic analysis, phenotypic evaluation, repeated-dose 90-day oral toxicity study, safety assessment

## Abstract

Probiotics, as defined by the World Health Organization, are live microorganisms that, when consumed in sufficient quantities, provide health benefits to the host. Although some countries have approved specific probiotic species for use in food, safety concerns may still arise with individual strains. *Lacticaseibacillus paracasei* subsp. *paracasei* NTU 101 (NTU 101), isolated from the gut of healthy infants, has demonstrated various probiotic effects and shown safety in a prior 28-day animal feeding study. To further verify its safety and mitigate potential risks, we performed a comprehensive genotypic and phenotypic safety evaluation in accordance with the European Food Safety Authority guidelines for safety assessment through whole genome sequencing and related literature. In this research, minimum inhibitory concentration testing identified NTU 101’s resistance to chloramphenicol; however, subsequent gene analysis confirmed no associated risk of resistance. Assessments of safety, including biogenic amine content, hemolytic activity, mucin degradation, and D-lactic acid production, indicated a low level of risk. Additionally, a repeated-dose 90-day oral toxicity study in Sprague-Dawley rats revealed no toxicity at a dose of 2000 mg/kg body weight, further supporting the strain’s safety for consumption. Based on these comprehensive analyses, NTU 101 is considered safe for regular consumption as a health supplement.

## 1. Introduction

*Lactobacilli* are one of the most prominent groups of probiotics, widely known for their long history of consumption and their general recognition as safe for human use [1,2]. However, the possibility of unforeseen safety concerns cannot be entirely excluded. Factors such as the strain’s growth conditions, the ingredients and methods used in the production of bacterial powder, and storage conditions may result in genetic changes, altered gene expression, and shifts in metabolic products [3,4]. While certain bacterial species are traditionally considered safe, the use of non-traditional delivery formats and increased dosages can introduce potential safety risks. Additionally, some strains, although deemed safe, originate from environments such as human or animal feces, raising questions about their safety under these circumstances [4].

A report by the Food and Agriculture Organization (FAO) and the World Health Organization (WHO) emphasizes the importance of safety considerations for probiotic strains [5]. According to the report, probiotics can pose four potential adverse effects: systemic infections, harmful metabolic activities, excessive immune stimulation in susceptible individuals, and gene transfer [6]. The FAO/WHO working group recommends several tests for probiotic safety confirmation: (1) determination of antibiotic resistance patterns; (2) assessment of specific metabolic activities, such as D-lactate production and bile salt deconjugation; (3) monitoring side effects during human studies; (4) epidemiological surveillance of adverse incidents in consumers; and (5) testing for toxin production, especially if the strain is known to produce mammalian toxins. If the strain belongs to a species with known hemolytic potential, hemolytic activity must also be assessed.

Various regulatory bodies have published lists of microorganisms approved for use in food, with the Qualified Presumption of Safety (QPS) list from the European Food Safety Authority (EFSA) being one of the most well-known. First released in 2007, the QPS list is regularly reviewed and updated by expert committees and includes microorganisms considered safe for consumption or use in food additive production (https://www.efsa.europa.eu/en/topics/topic/qualified-presumption-safety-qps: accessed on 4 August 2024). EFSA guidelines provide specific standards for microbial analysis, including the quality and methods for whole genome sequencing (WGS), offering a standardized approach to safety assessments [7]. This emphasizes the crucial role of microbial identification in evaluating safety. Recent advancements in microbial classification methods have enhanced the precision and consistency of taxonomic identification [8], leading to more reliable safety assessments.

*Lacticaseibacillus paracasei* subsp. *paracasei* (formerly *Lactobacillus paracasei* subsp. *paracasei*) NTU 101, isolated from human feces, exhibits strong acid and bile tolerance [9]. Previous studies have demonstrated its various probiotic benefits, including improvements in gut health [10], metabolic function [11,12,13], and immune modulation [14]. NTU 101 has been shown to exert anti-obesity effects in obese rats by modulating the gut microbiota, fatty acid oxidation, lipolysis, and adipogenesis [15]. It also helps alleviate allergic reactions associated with atopic dermatitis, thereby preventing its onset [16]. Clinical trials have revealed that daily intake of NTU 101 enhances intestinal peristalsis, immunity, and antioxidant capacity [17]. NTU 101 may promote peristalsis through the purinergic pathway, potentially preventing constipation-related irritable bowel syndrome [18].

As a species included in the QPS list, *Lacticaseibacillus paracasei* has already demonstrated sufficient safety. In previous studies, NTU 101 has passed multiple safety tests, including the bacterial reverse mutation test, cell chromosomal aberration test, rodent peripheral blood micronucleus test, and a 28-day oral toxicity assay [19]. The objective of this study is to follow the WHO/FAO safety assessment criteria and EFSA guidelines [7,20], using integrated phenotypic and genotypic analyses to confirm the safety of NTU 101 at the strain level. Building on the results of the 28-day oral toxicity assay, a repeat-dose 90-day oral toxicity study was conducted to further assess the long-term safety of NTU 101 under dietary exposure. This study aimed to evaluate the safety of NTU 101 when consumed over an extended period at various intake levels, helping to establish the range and flexibility of its use as a food ingredient.

## 2. Materials and Methods

### 2.1. Bacterial Strains and Growth Conditions

NTU 101 is stored long-term in glycerol cryotubes at −80 °C. The strain was also deposited in the German Collection of Microorganisms and Cell Cultures (DSMZ) under accession number 28047. For strain activation, the cryopreserved cells are streaked on De Man–Rogosa–Sharpe (MRS) (BD Difco, Franklin Lakes, NJ, USA) agar plates and incubated at 37 °C for 48–72 h. The reference strains used in the hemolysis activity assay and mucin degradation activity were purchased from the Bioresource Collection and Research Center (BCRC) in Hsinchu, Taiwan. These strains include *L. paracasei* subsp. *paracasei* BCRC 17002, *L. paracasei* subsp. *paracasei* BCRC 12248^T^, *L. paracasei* subsp. *paracasei* BCRC 14023, and *L. rhamnosus* BCRC 16000. These strains are preserved and activated in the same manner as NTU 101.

### 2.2. Taxonomic Identification Through Phenotypic and Genotypic Analysis

For phenotypic identification, Gram staining was initially performed to determine the Gram stain type of the strain. The microscopic morphology of the colonies was then examined to assess their appearance. Additionally, the API 50 CHL Identification System (Biomérieux, Craponne, France) was employed for phenotypic characterization. In terms of genotypic identification, performed according to the standards outlined by Resico and Trujillo (2024) [8], the 16S rDNA sequence obtained through Sanger sequencing was used to assess sequence similarity for strain identification, utilizing a Basic Local Alignment Search Tool for nucleotide (BLASTn) search in the NCBI database.

For WGS, genomic DNA from NTU 101 was extracted using overnight-cultured liquid samples. The extraction was performed according to the manufacturer’s instructions using the Quick-DNA™ Fungal/Bacterial Miniprep Kit (Zymo Research, Irvine, CA, USA). The whole genome sequencing was performed using a MinION Mk1B device (Oxford Nanopore, Oxford, UK) with an R9.4.1 flow cell (FLO-MIN106D) and SQK-LSK109 Ligation Sequencing Kit (Kit 9 chemistry), which were used according to the Nanopore protocol. Library preparation was carried out using KAPA Hyper Prep (Roche, Rotkreuz, Switzerland) and KAPA HyperPure Beads (Roche, Rotkreuz, Switzerland). The sequencing data processing and assembly were performed according to the procedures described in Murigneux et al. (2021) [21], using Flye (v2.9, https://github.com/mikolmogorov/Flye, accessed on 1 July 2022) as the assembler, which simultaneously assembles both chromosomes and plasmids. Further polishing of the assembly was performed using Homopolish. (v0.3.3, https://github.com/ythuang0522/homopolish, accessed on 1 July 2022).

Genome completeness was assessed using CheckM (v0.9.7). To confirm the genetic taxonomy, the average nucleotide identity (ANI) was calculated using the Prokaryotic Genome Annotation Pipeline (PGAP) (version 6.7, 2024-07-18.build7555), with default command-line parameters. Additionally, digital DNA–DNA hybridization (dDDH) was assessed using the Genome-to-Genome Distance Calculator (GGDC) implemented in the Type Strain Genome Server (TYGS, https://tygs.dsmz.de/, accessed on 22 October 2024) to determine the genomic similarity of the strain. The Newick tree format was generated by the TYGS service and the phylogenetic tree was constructed using Mega11 (version 11.0.13, https://www.megasoftware.net/, accessed on 1 August 2024).

### 2.3. Gene Prediction and Annotation

Gene prediction and annotation for NTU 101 were similarly performed using the PGAP, with default command-line parameters. The classification of protein clusters of orthologous groups (COGs) was conducted using COGclassifier (v1.0.5, https://github.com/moshi4/COGclassifier, accessed on 14 August 2024), which analyzed the PGAP-annotated protein sequences with default parameters. The analysis utilized a reverse position-specific BLAST search against the NCBI conserved domain database (2020 update). Carbohydrate-Active enZYmes Database (CAZy, http://www.cazy.org/, accessed on 8 August 2024) gene annotation was performed using the dbCAN server (HMMdb v12) (https://bcb.unl.edu/dbCAN2/, accessed on 30 July 2024). Annotation of the Kyoto Encyclopedia of Genes and Genomes (KEGG) was conducted using the hidden Markov model (HMM) profile (ver. 2024-05-01, KEGG release 110.0) and analyzed with the KofamKOALA server (https://www.genome.jp/tools/kofamkoala/, accessed on 30 July 2024), which utilizes the kofamScan 1.3.0 tool.

The detection of mobile genetic elements was performed using MobileElementFinder (https://cge.food.dtu.dk/services/MobileElementFinder/, accessed on 31 July 2024) to assess the potential for horizontal gene transfer of antimicrobial resistance genes and virulence factors. For virulence factor analysis, protein sequences from the full dataset of the Virulence Factor Database were downloaded (version 26-07-2024, http://www.mgc.ac.cn/VFs/download.htm, accessed on 30 July 2024). The searching algorithm of BLAST version 2.16.0+, including BLASTn, BLASTp, and BLASTx, was used to search the protein sequences of NTU 101 annotated by PGAP and annotation tools to identify potential pathogenic and virulence factor genes.

### 2.4. Antibiotic Susceptibility Assay and Resistance Genes Prediction

The phenotypic analysis of antimicrobial susceptibility was performed according to ISO 10932:2010 [22], in accordance with EFSA guidelines [20,23], to determine the minimal inhibitory concentration (MIC). The MIC values were tested and compared with the EFSA cut-off value for *Lacticaseibacillus paracasei* to determine susceptibility.

Antibiotic resistance genes (ARGs) were screened using the following databases and tools. The Comprehensive Antibiotic Resistance Database (CARD) version 3.2.9 (https://card.mcmaster.ca/home, accessed on 26 July 2024) was used, employing the Resistance Gene Identifier (RGI) version 6.0.3 to analyze the NTU 101 WGS data. For additional screening, ResFinder version 4.5.0 (http://genepi.food.dtu.dk/resfinder, accessed on 26 July 2024) was used with specific analysis conditions. AMRFinderPlus version 3.12.8 (https://github.com/ncbi/amr, accessed on 30 July 2024) was used to analyze the NTU 101 protein sequences annotated by PGAP, following the usage instructions provided.

### 2.5. Analysis of Biogenic Amine

The analysis of biogenic amines (BAs) in fermentation broths was performed using high-performance liquid chromatography (HPLC). The conditions and gradient for HPLC were adapted from the methods described by Sang et al. (2020) [24] and Ma et al. (2021) [25]. The biogenic amines analyzed, including putrescine, cadaverine, tryptamine, histamine, tyramine, spermidine, and spermine, were purchased from Dr. Ehrenstorfer (LGC Standards, Teddington, U.K.), while 2-phenylethylamine was obtained from Sigma (St. Louis, MO, USA). Dansyl chloride (ACROS, Geel, Belgium) was used for the derivatization of these biogenic amines, following the method described by Eerola et al. (1993) [26]. This derivatization process was applied to standard BA samples as well as to BAs present in three types of fermentation media, including MRS broth, milk, and soymilk, all fermented by NTU 101.

### 2.6. Hemolytic Activity Assay

Following the methods described by Casarotti et al. (2017) [27], activated NTU 101, reference lactic acid bacteria strains, and the positive control strain *S. aureus* BCRC 12154 were cultured. The bacterial suspensions were inoculated onto CMP^TM^ Columbia Agar (BD Difco, USA) with 5% defibrinated sheep blood using a four-quadrant streaking method. The plates were incubated at 37 °C under both aerobic and anaerobic conditions for 48 h. After incubation, the appearance of the colonies and the surrounding areas on the plates were observed. According to the standards set by Buxton (2005) [28], α-hemolysis was indicated by a green or brown discoloration around the colonies, β-hemolysis was indicated by a clear zone surrounding the colonies, and γ-hemolysis was indicated by no change in the coloration around the colonies.

### 2.7. Mucin Degradation Activity

Following the methods described by Zhou et al. (2001) [29], type III mucin from porcine stomach (PSM) (Sigma, USA) was pretreated. Two types of agar plates were prepared: 0.5% *w/v* PSM with or without 3% *w/v* glucose, referred to as PSMG and PSM agar, respectively, according to the formulation by Zhou et al. (2001). Activated NTU 101, reference lactic acid bacteria strains, and the positive control strain *S. enterica* subsp. *enterica* BCRC 10747 were each cultured in liquid form. A 10 µL drop of each bacterial suspension was placed in the six quadrants of the pre-prepared PSMG and PSM agar plates. The plates were then incubated at 37 °C under aerobic conditions for 72 h. After incubation, the plates were stained for 30 min with 0.1% *w/v* amido black (Sigma, USA) dissolved in 3.5 M acetic acid and subsequently washed with 1.2 M acetic acid. Colonies that turned deep blue after staining and exhibited a clear zone around them were considered positive for mucin degradation activity. If no colony growth was observed and the surrounding area remained light blue after staining, the result was classified as negative for mucin degradation activity.

### 2.8. Translocation on Intestinal Epithelial Cells

In this study, we examined whether NTU 101 bacterial powder can translocate across a monolayer of intestinal epithelial cells under conditions of barrier dysfunction induced by hydrogen peroxide (H_2_O_2_), following the approach described by Fatmawati et al. (2020) [30]. C2BBel BCRC 60182 cells, a clone of human colon adenocarcinoma cells (Caco-2, ATCC HTB-37), were seeded into transwell apical chambers (8.0 μm pore size) at a density of 4 × 10^4^ cells per well and cultured with Dulbecco’s modified Eagle medium (DMEM), supplemented with 20% fetal bovine serum and 0.01 mg/mL holo-transferrin (Sigma, USA).

We used a Millicell ERS-2 voltohmmeter (Millipore, Darmstadt, Germany) to monitor transepithelial electrical resistance (TEER) values throughout the culture period. When the TEER value exceeded 300 Ω × cm^2^, the monolayer was deemed suitable for use in experiments. H_2_O_2_ was used to induce intestinal barrier dysfunction by co-incubating for 2 to 3 h. Then NTU 101 bacterial suspensions (10^8^ CFU/mL) were added to both untreated cells and cells with H_2_O_2_-induced dysfunction for 1 h. Following incubation, the cell culture medium was centrifuged at 8000–10,000× *g*, and an aliquot was plated on MRS agar. Colony growth of NTU 101 was assessed visually after 48 to 72 h of incubation. The absence of colony growth indicated that bacterial translocation did not occur under conditions of normal epithelial barrier function.

### 2.9. D/L-Lactic Acid Production

Activated NTU 101 was subcultured at a 1% (*v*/*v*) concentration into sterilized MRS liquid medium in test tubes. The cultures were incubated at 37 °C under anaerobic conditions for 24 h. For lactic acid analysis, fermentation samples were prepared according to the method described by Kim et al. (2022) [31]. After incubation, the fermentation broth was centrifuged at 5000× *g* and 4 °C for 10 min to obtain the supernatant. The supernatant was then filtered through a 0.22 μm filter to obtain the filtrate for subsequent analysis. The concentrations of D- and L-lactic acids were measured using the D-Lactic Acid (D-lactate) (Rapid) Kit and L-Lactic Acid (L-Lactate) Assay Kit (Megazyme, Bray, Co. Wicklow, Ireland) following the manufacturer’s instructions.

### 2.10. Bile Salt Hydrolase Activity

The bile salt hydrolase (BSH) activity was assessed using the method described by Dashkevicz and Feighner (1989) [32]. Activated NTU 101 and the positive control strain *L. acidophilus* BCRC 10695^T^ were streaked onto MRS agar plates and MRS agar plates containing 0.5% (*w*/*v*) taurodeoxycholic acid (TDCA) (Sigma, USA). The plates were incubated at 37 °C under anaerobic conditions for 48 h. After incubation, the colonies and the surrounding areas were observed. A positive BSH activity was indicated by the presence of a white precipitate either on the colonies or in the surrounding area. If no color changes or colonies formation were observed, the result was classified as negative for BSH activity.

### 2.11. Repeated-Dose 90-Day Oral Toxicity Study in Rats

A repeated-dose 90-day oral toxicity study was conducted in compliance with Good Laboratory Practice for Nonclinical Laboratory Studies (21 CFR Part 58), FDA, USA, 1987; OECD Principles on Good Laboratory Practice, ENV/MC/CHEM (98) 17, 1998; and General requirements for the competence of testing and calibration laboratories (ISO/IEC 17025), International Organization for Standardization, 2005 [33]. All procedures involving animal handling were reviewed and approved by the Institutional Animal Care and Use Committee (IACUC) of Super Laboratory Co., Ltd. (New Taipei City, Taiwan) under approval number 105-91.

The test article used in this study was NTU 101 powder (brand name: Vigiis 101-LAB, containing 1 × 10^11^ CFU/g of NTU 101). Forty male and forty female Sprague-Dawley (SD) rats were obtained from BioLASCO Taiwan Co., Ltd. (Taipei, Taiwan) and were subjected to 12 h light/dark cycle with a maintained relative humidity of 55 ± 15% and a temperature of 22 ± 3 °C. Feed (MFG; Oriental Yeast Co., Tokyo, Japan) and sterile reverse osmosis (RO) water were provided with a water bottle. The test article was administered to the low-, medium-, and high-dose groups in a dose of 500 mg/kg B.W., 1000 mg/kg B.W., and 2000 mg/kg B.W., which were 30-, 60-, and 120-fold the human recommended daily intake (1000 mg/60 kg B.W.), respectively. There were ten male and ten female SD rats in each group. The test article was weighted daily, dissolved in an adequate amount of reverse osmosis water, and mixed evenly.

All analyses and records were conducted in accordance with the previously mentioned guidelines and standards, including daily clinical observations to monitor morbidity and mortality signs in all rats. Body weight was measured weekly, and food consumption was also recorded weekly. Hematology, clinical biochemistry, pathology, gross necropsy, and histopathology were performed following the procedures and parameters outlined in these guidelines and standards. Blood, urine, and organ samples were collected for analysis and data recording. For clinical chemistry analysis, blood samples were analyzed using an automated analyzer (7070 Autoanalyzer, Hitachi, Tokyo, Japan). For urinalysis, the collected urine samples were analyzed using a compact urine analyzer (PU-4010, ARKRAY Core Laboratory, Kyoto, Japan). For histopathological examination, the fixed organs of the control group and high-dose group were subjected to tissue section for histopathological examination. These fixed organs were dehydrated, clarified, infiltrated with paraffin, and embedded after trimming, forming paraffin tissue blocks, which were cut into 2 μm thickness of a tissue slice using a paraffin tissue slicing machine (Leica, Heidelberger, Germany) and stained with hematoxylin and eosin (H&E). If the test article treatment-related changes were observed in a particular organ or tissue in the high-dose group, then the examination should be extended to the organ or tissues of the medium-dose group and low-dose group.

For the 90-day oral toxicity study, all data were expressed as mean and standard deviation (S.D.). The SPSS statistical software (https://www.ibm.com/products/spss-statistics, accessed on 1 August 2024) was used for the analysis. The body weight, feed intake, organ weight, hematology, and clinical biochemistry analysis were carried out using one-way ANOVA followed by Duncan’s multiple range test to determine the significant difference between the treatment and control group. A significant difference was defined as a *p* < 0.05.

## 3. Results and Discussions

### 3.1. WGS, Taxonomic Identification, and Annotation Results

Phenotypic identification of NTU 101 revealed it to be rod-shaped under microscopic observation and classified as a Gram-positive bacterium (Figure S1). Strain identification, based on carbon fermentation characteristics using API 50 CHL, confirmed it as *Lacticaseibacillus paracasei* subsp. *paracasei* (Table S1). Phylogenetic analysis of the 16S rDNA sequence obtain from Sanger sequencing of NTU 101 showed that its closest relatives were *L. paracasei* R094^T^, *L. paracasei* NBRC 15889^T^, *L. paracasei* subsp. *tolerans* NBRC 15906^T^, *L. chiayiensis* BCRC 81062^T^, *L. zeae* RIA 482^T^, and *L. casei* DSM 20011^T^, all with sequence similarities greater than 99%, a common threshold for species delineation.

The complete genome sequence of NTU 101 has been submitted to the NCBI GenBank under accession number GCA_002901165.3 and is summarized in Table S2. This sequence was compared to the genome of *L. paracasei* subsp. *paracasei* JCM 8130, the type strain of *L. paracasei* subsp. *paracasei*. The NTU 101 genome is 3,061,587 base pairs (bp) in length with a GC content of 46.5%, consisting of a single circular chromosome of 3,010,957 bp and a plasmid of 50,630 bp. Gene annotation using PGAP revealed 2956 genes, including 2879 coding DNA sequences (CDSs). In the analysis of COGs, 82.18% (2287 out of 2783) of the sequences were classified into COG functional categories, with the highest number of genes associated with category G (carbohydrate transport and metabolism) (Figure S2).

To assess the quality of genome sequencing and assembly, we used CheckM, a standard tool for evaluating microbial genome completeness and contamination [34]. A genome completeness greater than 90% and contamination below 5% are considered thresholds for high-quality genomes [34,35]. The NTU 101 genome met these criteria, with a CheckM completeness of 91.88% and contamination of 4.13%, comparable to the JCM 8130^T^ genome.

The average nucleotide identity (ANI) between NTU 101 and JCM 8130^T^ was calculated using PGAP’s built-in ANI tool, resulting in a similarity of 98.682%. Additionally, results from the TYGS analysis yielded a digital DNA–DNA hybridization (dDDH) value of 88.2% similarity with JCM 8130^T^, using the *d4* formula (formula 2 in GGDC). Both values exceed the accepted species boundaries of ANI greater than 95–96% and dDDH above 70% [8,36,37]. Furthermore, the TYGS analysis indicated that both the phylogenetic tree based on 16S rDNA sequences and the phylogenomic tree based on whole genome sequences showed that NTU 101 is more closely related to the *L. paracasei* subsp. *paracasei* type strains (JCM 8130^T^ and ATCC 25302^T^) (Figures S3 and S4). Based on these findings, NTU 101 is confirmed to belong to the species *Lacticaseibacillus paracasei* subsp. *paracasei*.

### 3.2. Antibiotic Resistance

The results of the MIC and genotypic analysis for NTU 101 are presented in Table 1. With the exception of chloramphenicol, all MIC values were below the cut-off limits established by the EFSA for *Lacticaseibacillus paracasei* species [20]. These findings are consistent with the MIC results for BCRC 12248^T^ (Table 1) and align with previous reports in the literature [38,39]. According to EFSA guidelines, when a strain exhibits antibiotic resistance, it is essential to identify the genetic determinants of this resistance to rule out the possibility of acquired resistance [23].

To analyze potential antibiotic resistance genes, we compared NTU 101 against three continuously updated databases. A search using RGI against the CARD database identified a “Strict” hit, which detect previously unknown variants of known antibiotic resistance genes (ARGs) [40] for the *qacJ* gene, which showed 38% sequence identity, well below the EFSA threshold of 80%. Further analysis using BLASTp in the NCBI RefSeq protein database confirmed that *qacJ* encodes a multidrug efflux pump from the SMR transporter family, specifically associated with the EmrE conserved domain. Previous studies have shown that the EmrE transporter in *Escherichia coli* does not confer resistance to chloramphenicol [41]. Additionally, this gene was detected in other *Lacticaseibacillus* species within the BLASTp results, suggesting that the EmrE-encoding gene is likely intrinsic to *Lacticaseibacillus* and is unlikely to be transferable.

Moreover, we identified 284 “Loose” hits, which detect distant homologs of ARGs or emerging threats [40], but none were considered potential risk genes after filtering for an 80% identity threshold. We also used ResFinder and AMRfinderPlus to detect acquired antimicrobial and disinfectant resistance genes, with neither method detecting any resistance genes. Additionally, analysis using MobileElementFinder revealed no mobile genetic elements carrying resistance genes. Based on these analyses, NTU 101 can be considered safe with respect to antibiotic resistance.

### 3.3. Biogenic Amines Production

We next analyzed the biogenic amine content in the fermentation broth of NTU 101 cultivated in MRS medium, milk, and soymilk using HPLC. The results revealed a noticeable increase in biogenic amine levels. In the MRS medium, cadaverine was detected at 4.18 ppm. During milk fermentation, putrescine (4.54 ppm), cadaverine (2.85 ppm), 2-phenylethylamine (3.79 ppm), and spermine (22.47 ppm) were identified. In contrast, soymilk fermentation produced histamine (2.44 ppm) and tyramine (12.73 ppm) (Table 2).

Biogenic amines are primarily formed by bacterial decarboxylation of amino acids, a process catalyzed by decarboxylase [42]. To investigate this further, we analyzed the NTU 101 protein sequences using the KofamKOALA gene function annotation tool available on the KEGG website. The results, presented in Table 3, showed no genes encoding histidine decarboxylase or tyrosine decarboxylase, which are responsible for histamine and tyramine production, respectively, in the NTU 101 genome. Similarly, no genes responsible for the decarboxylation of 2-phenylethylamine, spermine, or tyramine were detected. Additionally, no other enzymes involved in these synthetic pathways were identified.

In the gene annotation results obtained from PGAP, NTU 101 was found to contain the gene encoding L-ornithine decarboxylase [EC: 4.1.1.17], which is commonly observed in *L. paracasei* strains and other *Lacticaseibacillus* species within the BLASTp search of the RefSeq protein database. L-ornithine decarboxylase catalyzes the conversion of L-ornithine to putrescine. This enzyme has been previously reported in the *L. paracasei* genome [43,44,45]. Furthermore, we identified intact genes encoding the four subunits of the channel protein PotABCD, which transports putrescine [46,47]. Additionally, a gene encoding putative lysine decarboxylase [EC 4.1.1.18], responsible for producing cadaverine from lysine, was also identified, though conserved domain analysis suggested it may not correspond to this enzyme.

The genes encoding synthetic enzymes related to certain biogenic amines, including 2-phenylethylamine, tyramine, and spermine, were not detected, which aligns with findings from previous studies [48,49]. It has been hypothesized that an unidentified enzyme with amino acid decarboxylase activity may exist, warranting further investigation into the enzymes involved in biogenic amine production.

Lactic acid bacteria often metabolize amino acids to generate energy or as a survival mechanism in low-pH environments [50]. According to a report by the EFSA [51], fermented dairy products are known to contain biogenic amines, with average levels of histamine, tyramine, putrescine, and cadaverine ranging from 0.3 to 65.1, 0.3 to 335, 0.7 to 449, and 1.9 to 628 ppm, respectively. Acid curd cheese typically contains higher concentrations of biogenic amines (51.3 to 628 ppm) compared to yogurt (0.5 to 3.2 ppm). Similar levels of biogenic amines are found in fermented soy products, though concentrations vary depending on the food type [52].

Although spermine levels in fermented dairy products are generally low (0.02 to 0.8 ppm), it is present in higher concentrations in other foods, such as lean beef (27.3 to 36.4 ppm) [53]. In fermented soy products, spermine levels typically range from 1.3 to 242 ppm [52]. The biogenic amine content produced by NTU 101 is relatively low compared to that found in fermented or general foods, suggesting a minimal risk of harm [52,53]. Thus, it is unlikely that the biogenic amines produced by NTU 101 in fermented foods pose any significant health risk.

### 3.4. Virulence Factor and Hemolysis Activity

We performed a BLAST search of the NTU 101 genome against sequences from the VFDB to identify potential virulence genes. The VFDB dataset is divided into DNA and protein categories, and our analysis utilized the full datasets from both categories. These datasets encompass known and predicted virulence factor genes associated with pathogenicity. To begin, we applied the EFSA criteria (identity > 80%, coverage > 70%) to analyze the results from both the DNA and protein databases. After screening, no potential virulence genes were detected. Even when the identity threshold was lowered to greater than 70%, with coverage still above 70%, the DNA dataset analysis did not reveal any virulence genes. However, the protein database analysis identified four potential virulence genes. These four protein sequences were further examined using BLASTp in the NCBI RefSeq protein database, revealing that these genes are commonly found in *Lacticaseibacillus species*.

**Figure 1:**
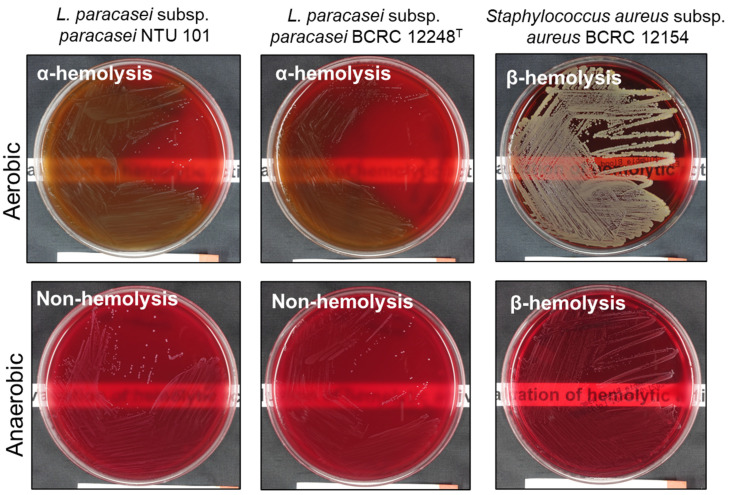
Hemolysis type test by 5% sheep blood agar assay of each strain, cultured under different anaerobic conditions. The bottom piece of paper with printed text shows the difference in transparency of plates of different strains.

Phenotypic and genotypic methods were employed for hemolytic activity evaluation. Phenotypic analysis showed that NTU 101 and *L. paracasei* subsp. *paracasei* BCRC 12248^T^ exhibited α-hemolysis under aerobic conditions. However, no hemolysis (gamma hemolysis) was observed when NTU 101 and BCRC 12248^T^ were cultured under anaerobic conditions (Figure 1). Similarly, two other *L. paracasei* subsp. *paracasei* strains (BCRC 14023 and BCRC 17002) and *Lacticaseibacillus rhamnosus* BCRC 16000 also showed α-hemolysis under aerobic conditions, with no hemolysis under anaerobic conditions. This aligns with previous studies that report *Lactobacillus* species typically exhibit α-hemolysis on blood agar [54]. This characteristic has been consistently documented in various reports on *L. paracasei* strains, such as *L. paracasei* Lpc-37 [55] and L2 [56]. These findings suggest that α-hemolysis is a common trait among lactic acid bacteria (LAB), particularly in *L. paracasei* strains.

Our VFDB search identified three hemolytic toxin-coding genes in the NTU 101 genome, which may belong to the hemolysin III family (one gene) and the hemolysin family (two genes) (Table 4). BLASTp searches in the RefSeq protein database indicated that these proteins are highly conserved across *Lacticaseibacillus*, encoded by *YafA* and *TlyC* (Table 5). Although the *YqfA* gene is commonly found in the *Lacticaseibacillus* genus [57], no safety concerns have been reported in relation to this gene. Additionally, BLASTp analysis confirmed the presence of the *TlyC*-encoded hemolysin protein family in other *L. paracasei* strains and other *Lacticaseibacillus* species. Importantly, the phenotypic analysis of NTU 101 on blood agar demonstrated no β-hemolysis. Furthermore, no safety concerns related to hemolysis have been reported in previous studies on *L. paracasei*. Thus, we conclude that NTU 101 poses no significant risk of hemolysis.

### 3.5. Mucin Degradation and Intestinal Mucosal Barrier

**Figure 2:**
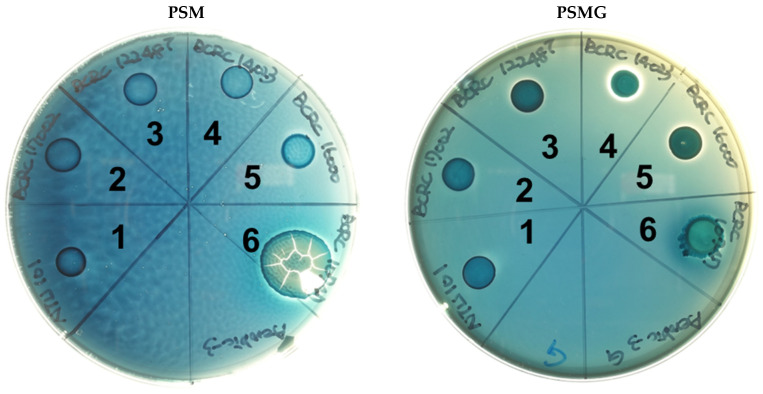
The results of the mucin degradation activity assay of NTU 101 and reference strains. PSM is the medium with mucin as the sole carbon source. PSMG is the PSM with additional glucose. Each number represents the following strain: (1) *L. paracasei* subsp. *paracasei* NTU 101. (2) *L. paracasei* subsp. *paracasei* BCRC 17002. (3) *L. paracasei* subsp. *paracasei* BCRC 12248^T^. (4) *L. paracasei* subsp. *paracasei* BCRC 14023. (5) *L. rhamnosus* BCRC 16000. (6) *S. enterica* subsp. *enterica* BCRC 10747 (mucin degradation activity-positive strain).

Mucin degradation is an undesirable characteristic in probiotics due to its potential to compromise the intestinal mucosal barrier, which plays a crucial role in host defense. Disruption or damage to this protective mucin layer can lead to adverse effects [29]. To assess whether NTU 101 possesses mucin-degrading activity, we conducted a mucin hydrolysis test. NTU 101 did not grow on PSM-only plates, and no mucin degradation was observed (Figure 2). In contrast, the positive control strain, *Salmonella enterica* subsp. *enterica* BCRC 10747, grew normally (Table 6). Similarly, previous studies of *Lactobacillus* and *Bifidobacterium* species, which are commonly used probiotic strains, also reported no mucin-degrading activity [29,58,59].

Microbes that degrade mucin typically possess glycosyl hydrolases (GHs), enzymes that cleave specific glycan linkages. According to Glover et al. (2022), the key GHs involved in mucin degradation include GH33, GH29, GH95, and GH20/35 [60]. Whole-genome analysis of NTU 101 using the CAZy database revealed that it contains genes associated with GH29 and GH20/35 but lacks those for GH33 and GH95 (Table 7). As a result, NTU 101 does not have the complete genetic capability required for mucin degradation, which aligns with the results of the mucin degradation phenotype experiment. To further evaluate the potential impact of NTU 101 on the intestinal barrier, we utilized the C2BBe1 intestinal epithelial cell line to establish a monolayer membrane model mimicking the intestinal barrier. No translocation of NTU 101 was observed in this model with intact barrier function. In conclusion, NTU 101 does not degrade mucin nor compromise the integrity of the intestinal mucosal barrier.

### 3.6. D/L-Lactic Acid Production and Bile Salts Hydrolase Activity

The production of L- and D-lactic acid by NTU 101 was assessed, revealing that 96.3% of the lactate produced was the L-enantiomer. D-lactic acid is generated from pyruvate via the enzyme D-lactate dehydrogenase. Gene annotation results for NTU 101 identified an enzyme with D-lactate dehydrogenase activity, specifically D-2-hydroxyacid dehydrogenase, suggesting that the D-lactic acid present in the fermentation broth is produced by NTU 101. D-lactic acidosis, a rare form of lactic acidosis, is most commonly observed in patients with short bowel syndrome. High levels of D-lactate in the bloodstream can lead to neurological symptoms such as delirium, ataxia, and dysarthria [61]. Although symptomatic patients typically exhibit elevated D-lactate levels, the risk of D-lactic acidosis in healthy individuals is considered low [62]. Compared to other lactic acid bacteria, NTU 101 produces a relatively low proportion of D-lactate. For example, *Limosilactobacillus reuteri* NCIMB 3053 produces 54.5% D-lactate, and *Lactobacillus delbrueckii* ATCC 11842 produces 52.2% [63]. In contrast, NTU 101’s D-lactate proportion is comparable to other GRAS (generally recognized as safe) strains, such as *Lacticaseibacillus rhamnosus* GG and *Bifidobacterium animalis* subsp. *lactis* BB-12, both of which produce less than 5% D-lactate [64,65].

**Figure 3:**
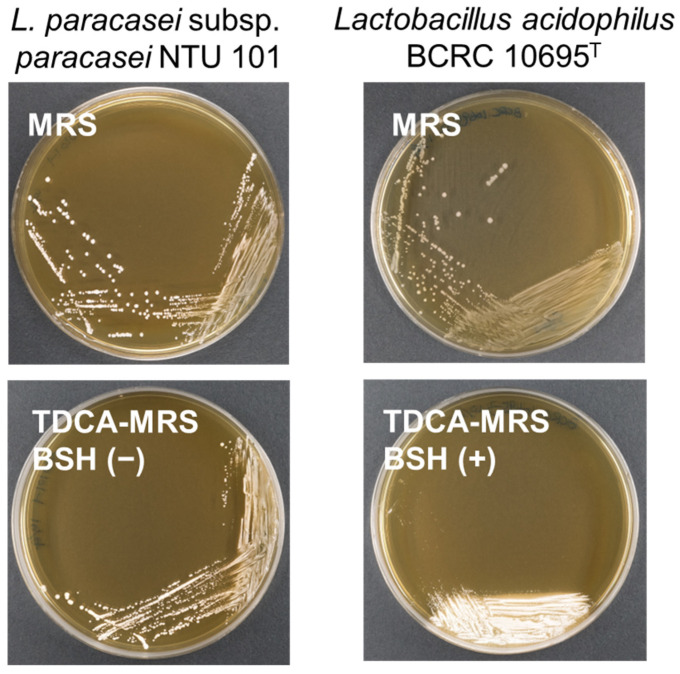
The results of BSH activity assay on MRS plates and 5% TDCA containing MRS plates (TDCA-MRS). The white precipitate beneath the colonies is deoxycholic acid produced by BSH hydrolysis of TDCA, indicating that *L. acidophilus* BCRC 10695^T^ has positive BSH activity. BSH activity of NTU 101 was shown to be negative.

Phenotypic experiments on NTU 101 indicated no bile salt hydrolase (BSH) activity when compared to the positive control group in TDCA MRS agar plates (Figure 3). This is consistent with findings from other studies on *Lactobacillus* (based on the previous taxonomy), where many species were also found to lack BSH activity [27,66]. TDCA is a bile salt conjugate of deoxycholic acid and taurine. Bacterial BSH activity breaks down conjugated bile salts, resulting in free bile acids, which are poorly soluble and form a white precipitate [32,67].

Despite the absence of observable BSH activity, a putative gene encoding BSH was identified through a protein sequence analysis of NTU 101. A BLASTp search against the RefSeq protein database revealed that this gene encodes a linear amide C-N hydrolase. Previous research by Zhang et al. (2009) [68] suggested that the linear amide C-N hydrolase of *Lactobacillus casei* Zhang exhibits BSH activity. Sequence alignment of the linear amide C-N hydrolase from NTU 101 with that of *L. casei* Zhang (accession no. ACC93573.1) showed 100% similarity, indicating that NTU 101 may possess BSH functionality. Additionally, research by Elkins et al. (2001) [69] demonstrated that BSH activity in LAB is related to bile salt transporters. However, limited research exists on *Lacticaseibacillus* in this context, leading us to speculate that NTU 101’s BSH activity may be influenced by bile salt transporter functionality.

### 3.7. Repeated-Dose 90-Day Oral Toxicity Study

The results of the 90-day oral toxicity study revealed that none of the rats in either the treatment or control groups exhibited any abnormal clinical signs throughout the study period (Table 8 and Table 9). All rats in the treatment groups demonstrated normal weight gain (Table 10). Ophthalmological examinations revealed no abnormalities in any of the rats across both the treatment and control groups.

At the conclusion of the study, clinical pathology assessments, including hematology, clinical biochemistry, and urinalysis were conducted. While some parameters showed statistically significant differences, all values fell within or were close to the normal reference range. All surviving rats were sacrificed, and the absolute weights of major organs were measured (Table 10). Significant differences were noted in the spleen weight of the male medium-dose group, the brain weight of the male high-dose group, and the liver weight of the female medium-dose group compared to the control group. However, these differences remained within the normal reference range (based on Super Laboratory historical values). No pathological lesions related to the test article were observed in any associated organs, indicating that these changes had no clinical relevance.

Necropsy examinations found no significant abnormalities in rats from either the treatment or control groups. Histopathological analysis also revealed no specific organ toxicity or pathological changes associated with the test article in rats from the high-dose and control groups.

Based on these findings, the no-observed-adverse-effect level (NOAEL) of NTU 101 powder for the 90-day repeated-dose oral toxicity study in rats was determined to be 2000 mg/kg body weight (equivalent to 2 × 10^11^ CFU/kg body weight), which is 120 times the recommended daily human intake (1000 mg/60 kg body weight, equivalent to 1 × 10^11^ CFU/60 kg body weight).

## 4. Conclusions

Based on integrated phenotypic and genotypic analysis, NTU 101 poses no risk of antibiotic resistance. This strain does not exhibit β-hemolysis and only demonstrates α-hemolysis under aerobic culturing conditions, which is a typical characteristic of *Lactobacillus* strains. Additionally, there are no previous reports of pathogenicity associated with NTU 101. While it contains a few genes that encode enzymes capable of producing biogenic amines, only low levels of biogenic amines are produced in certain fermented foods. Importantly, NTU 101 lacks markers for pathogenic or toxigenic gene variants. The primary lactate produced by this strain is L-lactic acid, with D-lactic acid constituting less than 5% of the total lactate content. Although NTU 101 possesses the BSH gene, phenotypic testing showed no activity. Furthermore, phenotypic and genotypic assessments of mucin degradation revealed that NTU 101 cannot degrade mucin. No translocation of NTU 101 across intestinal epithelial cells was observed, suggesting that the strain does not compromise the intestinal barrier. The results of a 90-day repeated-dose oral toxicity study, along with previous human clinical trials, indicated no adverse reactions, even at a dose of 1000 mg/kg. Based on these safety assessment data, *L. paracasei* subsp. *paracasei* NTU 101 is considered safe for use in food products. Further studies will build on these findings to analyze the uncharacterized traits of NTU 101 and potential, yet undiscovered, safety risks using transcriptomics, proteomics, and metabolomics.

## Figures and Tables

**Table 1:** Antibiotic resistance results of NTU 101 through genotypic and phenotypic analysis. Antimicrobial minimum inhibitory concentrations (MICs) results compare to *L. paracasei* subsp. *paracasei* BCRC 12248^T^.

Resistance Target	Phenotypic Analysis				Genotypic Analysis		
	MIC (mg/L)			Susceptibility of NTU 101 ^b^	CARD	ResFinder	AMRfinderPlus
	Cut-Off Value ^a^	NTU 101	BCRC 12248^T^				
Ampicillin	4	2	1	S	N.D. ^e^	N.D.	N.D.
Vancomycin	n.r. ^c^	-	-	- ^d^	N.D.	N.D.	N.D.
Gentamicin	32	4	4	S	N.D.	N.D.	N.D.
Kanamycin	64	64	64	S	N.D.	N.D.	N.D.
Streptomycin	64	64	64	S	N.D.	N.D.	N.D.
Erythromycin	1	0.5	0.25	S	N.D.	N.D.	N.D.
Clindamycin	4	0.5	0.25	S	N.D.	N.D.	N.D.
Tetracycline	4	4	2	S	N.D.	N.D.	N.D.
Chloramphenicol	4	8	8	R	N.D.	N.D.	N.D.
*Disinfecting agents*							
Benzalkonium chloride	-	-	-	-	Strict (*qacJ*)	N.D.	-

^a^ According to EFSA guidance [20]. ^b^ Susceptible (S): S ≤ established cut-off value (mg/L); resistant (R): R > established cut-off value (mg/L). ^c^ n.r.: not required. ^d^ not applicable. ^e^ N.D.: not detected.

**Table 2:** Determination of biogenic amines in different fermentation media produced by NTU 101.

Fermented Medium	Group ^a^	Biogenic Amines (ppm) ^b^							
		HIS	TYR	PUT	TRP	PHE	CAD	SPD	SPR
*MRS*	B	N.D. ^c^	2.76 ± 0.14	N.D.	16.13 ± 0.70	3.66 ± 0.67	2.49 ^d^	11.97 ± 0.54	3.67 ± 0.17
	S	N.D.	2.83 ± 0.05	N.D.	13.83 ± 0.09	3.25 ± 0.09	4.18 ± 0.17	8.41 ± 0.23	3.33
*Skim milk*	B	N.D.	N.D.	N.D.	N.D.	N.D.	2.78 ± 0.17	N.D.	N.D.
	S	N.D.	N.D.	N.D.	N.D.	N.D.	3.11 ± 0.36	N.D.	N.D.
*Low-fat milk*									
Sample 1	B	N.D.	N.D.	N.D.	N.D.	N.D.	N.D.	N.D.	N.D.
	S	N.D.	N.D.	N.D.	N.D.	N.D.	2.68 ± 0.08	N.D.	N.D.
Sample 2	B	N.D.	12.09 ± 11.03	N.D.	N.D.	N.D.	2.20	N.D.	N.D.
	S	N.D.	25.26 ± 19.19	4.54	N.D.	N.D.	2.85	N.D.	N.D.
Sample 3	B	N.D.	N.D.	N.D.	N.D.	2.65	N.D.	2.73 ± 0.12	12.42 ± 7.44
	S	N.D.	N.D.	N.D.	N.D.	3.79	N.D.	2.78 ± 0.02	22.47 ± 9.58
*Soymilk*									
Sample 1	B	N.D.	N.D.	5.61 ± 0.60	10.68 ± 1.28	N.D.	2.70 ± 0.09	N.D.	N.D.
	S	N.D.	N.D.	N.D.	7.36 ± 2.06	N.D.	3.36 ± 0.36	N.D.	N.D.
Sample 2	B	6.83 ± 1.04	N.D.	4.91	13.60 ± 1.39	N.D.	2.29 ± 0.47	N.D.	N.D.
	S	3.07 ± 1.79	N.D.	N.D.	N.D.	N.D.	2.56 ± 0.11	N.D.	N.D.
Sample 3	B	2.91 ± 0.27	4.18	N.D.	6.60 ± 1.97	N.D.	3.11 ± 0.57	8.78 ± 5.96	N.D.
	S	2.89 ± 0.05	12.73 ± 1.44	N.D.	N.D.	N.D.	2.57 ± 0.16	9.33 ± 4.51	N.D.
Sample 4	B	2.33	2.40 ± 0.12	N.D.	N.D.	N.D.	N.D.	9.03 ± 0.20	N.D.
	S	2.44	2.39 ± 0.16	N.D.	N.D.	N.D.	N.D.	8.08 ± 0.30	N.D.

^a^ B: blank; S: fermented sample. ^b^ HIS: histamine; TYR: tyramine; PUT: putrescine; TRP: tryptamine; PHE: 2-phenylethylamine; CAD: cadaverine; SPD: spermidine; SPR: spermine. ^c^ N.D.: not detected. ^d^ Where only one replicate provided a detectable signal, standard deviation was not calculated.

**Table 3:** Analysis of the corresponding decarboxylase-encoding genes for the generation of biogenic amines by NTU 101.

Biogenic Amine	Enzyme of Decarboxylation	KEGG Entry	Annotated Protein ID	BLASTp Hit/Putative Conserved Domain
Histamine	Histidine decarboxylase	K01590	Not found	
Tyramine	Tyrosine decarboxylase	K01592, K18933, K22329, K22330	Not found	
Putrescine	L-ornithine decarboxylase	K01581	PGAP_000721	L-ornithine decarboxylase/*PRK13578* (ornithine decarboxylase; provisional)
	Agmatinase	K01480	Not found	
Tryptamine	L-tryptophan decarboxylase	K01593, K22433	Not found	
2-phenylethylamine	Phenylalanine decarboxylase	K22427	Not found	
	L-tryptophan decarboxylase	K01593	Not found	
Agmatine	Arginine decarboxylase	K01583, K01584, K01585, K02626	Not found	
Cadaverine	L-lysine decarboxylase	K01582	PGAP_000762	TIGR00730 family Rossman fold protein (putative lysine decarboxylases)/*PpnN* (nucleotide transport and metabolism)
	D-ornithine/D-lysine decarboxylase	K23385	Not found	
Spermidine	Spermidine synthase	K00797, K24034	Not found	
	Carboxynorspermidine decarboxylase	K13747	Not found	
Spermine	Spermine synthase	K00802	Not found	

**Table 4:** The hemolysis type of each strain. *Staphylococcus aureus* BCRC 12154 acts as β-hemolysis positive strain.

		Hemolysis Type ^a^	
No.	Strain	Aerobic Condition	Anaerobic Condition
1	*L. paracasei* subsp. *paracasei* NTU 101	α	γ
2	*L. paracasei* subsp. *paracasei* BCRC 17002	α	γ
3	*L. paracasei* subsp. *paracasei* BCRC 12248^T^	α	γ
4	*L. paracasei* subsp. *paracasei* BCRC 14023	α	γ
5	*L. rhamnosus* BCRC 16000	α	γ
6	*S. aureus* BCRC 12154	β	β

^a^ α-hemolysis, a greenish discoloration and partial hemolysis of the red blood cells immediately surrounding colonies on blood agar plates. β-hemolysis, a sharply defined clear colorless zone of hemolysis surrounding colonies on blood agar plates. γ-hemolysis, a lack of hemolysis in the area surrounding colonies on blood agar plates.

**Table 5:** Genotypic analysis of the hemolysis-related genes in NTU 101.

	VFDB Predicted		BLAST Result
Predicted Protein ID	VF Category	Function	BLASTp Hits ^a^/Putative Conserve Domain
PGAP_000462	VFG038907	Hemolysin III	Hemolysin III family protein [*Lacticaseibacillus paracasei*]/*YqfA*
PAGP_000832	VFG038902	Hemolysin A	Hemolysin family protein [*Lacticaseibacillus paracasei*]/*TlyC*
PAGP_001231	VFG038900	Hemolysin A	Hemolysin family protein [*Lacticaseibacillus paracasei*]/*TlyC*

^a^ Search against NCBI RefSeq protein database using the protein sequences of NTU 101.

**Table 6:** Results of mucin degradation ability of each strain, *Salmonella enterica* subsp. *enterica* BCRC 10747 as a positive strain.

		PSM		PSMG	
No.	Strain	Mucin Degradation	Colony Formation	Mucin Degradation	Colony Formation
1	*L. paracasei* subsp. *paracasei* NTU 101	− ^a^	− ^b^	−	−
2	*L. paracasei* subsp. *paracasei* BCRC 17002	−	−	−	−
3	*L. paracasei* subsp. *paracasei* BCRC 12248^T^	−	−	−	−
4	*L. paracasei* subsp. *paracasei* BCRC 14023	−	−	−	+
5	*L. rhamnosus* BCRC 16000	−	−	−	+
6	*S. enterica* subsp. *enterica* BCRC 10747	−	+	−	+

^a^ +, indicates a halo around the colony. −, indicates no halo. ^b^ +, indicates colony growth. −, indicates no colony growth.

**Table 7:** Genotypic analysis results of mucin degradation-relative genes of NTU 101. A single CAZy family may encompass multiple enzymes with distinct catalytic activities or substrate specificities.

CAZy Family	Predicted Protein ID	Corresponding Enzyme ^a^
GH2	PGAP_001529	β-galactosidase [EC 3.2.1.23]
GH20	PGAP_001643	β-N-acetylhexosaminidase [EC 3.2.1.52]
		Endo-beta-N-acetylglucosaminidase [EC 3.2.1.96]
		β-galactosidase [EC 3.2.1.23]
GH29	PGAP_000883 PGAP_002109	α-L-fucosidase [EC 3.2.1.51]
GH33		Not found
GH35	PGAP_001638	β-galactosidase [EC 3.21.23]
		β-fucosidase [EC 3.2.1.-]
		Glycosidases group [EC 3.2.1.38]
GH95		Not found

^a^ The results of searching the CAZy database using the protein sequences of NTU 101.

**Table 8:** Changes in hematological parameters in male and female rats treated with NTU 101 powder via gastric gavage for 90 consecutive days ^a^.

	Dose			
	Control	Low Dose (500 mg/kg)	Medium Dose (1000 mg/kg)	High Dose (2000 mg/kg)
Male				
WBCs (10^3^/μL)	11.9 ± 2.3	11.4 ± 2.1	12.1 ± 1.6	11.1 ± 2.2
RBCs (10^6^/μL)	9.7 ± 0.5	9.8 ± 0.8	9.6 ± 0.4	9.9 ± 0.6
HB (g/dL)	16.9 ± 0.5	16.9 ± 0.9	16.6 ± 0.8	17.1 ± 1.2
HCT (%)	49.8 ± 1.2	49.6 ± 2.8	48.8 ± 1.9	50.3 ± 3.5
MCV (fL)	51.4 ± 2.2	50.7 ± 2.6	50.6 ± 1.5	50.8 ± 2.7
MCH (pg)	17.4 ± 0.6	17.3 ± 0.8	17.2 ± 0.4	17.3 ± 0.8
MCHC (g/dL)	33.9 ± 0.4	34.0 ± 0.5	34.1 ± 0.8	34.1 ± 0.6
PLT (10^3^/μL)	955.2 ± 118.7	1026.1 ± 118.9	1040.4 ± 121.8	1043.0 ± 121.6
Neutrophils (%)	23.2 ± 7.3	24.6 ± 9.4	22.9 ± 5.2	23.0 ± 8.0
LYMPHs (%)	69.1 ± 7.6	68.4 ± 10.4	69.8 ± 5.4	70.5 ± 7.9
Monocytes (%)	6.2 ± 1.1	5.3 ± 1.3	5.5 ± 1.0	5.2 ± 0.7 *
Eosinophils (%)	1.3 ± 0.3	1.6 ± 0.5	1.5 ± 0.6	1.1 ± 0.4
Basophils (%)	0.2 ± 0.1	0.2 ± 0.1	0.3 ± 0.1	0.2 ± 0.1
PT (s)	14.9 ± 3.2	14.1 ± 1.5	13.0 ± 2.7	13.5 ± 2.1
Female				
WBCs (10^3^/μL)	8.8 ± 2.0	8.1 ± 2.2	8.6 ± 2.0	7.6 ± 1.0
RBCs (10^6^/μL)	8.9 ± 0.3	9.0 ± 1.1	9.0 ± 0.5	8.9 ± 0.6
HB (g/dL)	16.2 ± 0.8	16.5 ± 1.6	16.3 ± 1.0	16.4 ± 0.9
HCT (%)	47.6 ± 2.0	48.5 ± 4.3	48.0 ± 2.5	48.3 ± 2.2
MCV (fL)	53.8 ± 2.1	53.9 ± 2.4	53.4 ± 1.4	54.3 ± 2.1
MCH (pg)	18.3 ± 0.7	18.3 ± 0.6	18.1 ± 0.3	18.4 ± 0.5
MCHC (g/dL)	34.0 ± 0.6	34.0 ± 0.5	33.9 ± 0.6	33.8 ± 0.8
PLT (10^3^/μL)	1017.4 ± 91.4	1011.8 ± 174.0	1026.4 ± 156.6	902.6 ± 96.4
Neutrophils (%)	14.0 ± 2.4	15.3 ± 3.9	13.1 ± 3.1	13.3 ± 3.8
LYMPHs (%)	79.2 ± 2.6	78.4 ± 4.9	80.3 ± 5.3	80.6 ± 3.7
Monocytes (%)	5.2 ± 1.0	4.9 ± 1.3	5.0 ± 2.3	4.6 ± 1.0
Eosinophils (%)	1.3 ± 0.5	1.2 ± 0.4	1.2 ± 0.5	1.2 ± 0.3
Basophils (%)	0.3 ± 0.1	0.3 ± 0.1	0.3 ± 0.2	0.2 ± 0.1
PT (s)	9.7 ± 0.2	9.6 ± 0.2	9.7 ± 0.1	9.6 ± 0.2

WBCs: white blood cells; RBCs: red blood cells; HB: hemoglobin; HCT: hematocrit; MCV: mean corpuscular volume; MCH: mean corpuscular hemoglobin; MCHC: mean corpuscular hemoglobin concentration; PLT: platelet count; LYMPHs: lymphocytes; PT: prothrombin time. ^a^ Data expressed as mean ± S.D., *n* = 10. * Significant difference from control group (*p* < 0.05).

**Table 9:** Serum biochemistry parameters in male and female rats treated with NTU 101 powder via gastric gavage for 90 consecutive days ^a^.

	Dose			
	Control	Low Dose (500 mg/kg)	Medium Dose (1000 mg/kg)	High Dose (2000 mg/kg)
Male				
Glucose (mg/dL)	242.5 ± 27.2	242.9 ± 51.3	229.5 ± 39.8	259.8 ± 51.3
BUN (mg/dL)	15.6 ± 2.1	14.1 ± 1.7	14.8 ± 1.1	15.4 ± 1.2
Creatinine (mg/dL)	0.70 ± 0.07	0.67 ± 0.05	0.67 ± 0.05	0.68 ± 0.04
AST (U/L)	97.4 ± 21.4	83.1 ± 8.3	90.1 ± 18.5	111.7 ± 50.0
ALT (U/L)	44.2 ± 22.0	34.7 ± 4.3	36.5 ± 7.8	58.2 ± 42.8
Total protein (g/dL)	6.7 ± 0.3	6.9 ± 0.2	6.6 ± 0.2	6.8 ± 0.4
Albumin (g/dL)	4.6 ± 0.2	4.6 ± 0.1	4.5 ± 0.1	4.6 ± 0.2
ALP (U/L)	95.5 ± 21.0	93.0 ± 20.8	94.6 ± 21.7	105.9 ± 25.1
γ-GT (U/L)	<2.0	<2.0	<2.0	<2.0
Cholesterol (mg/dL)	67.4 ± 11.0	72.4 ± 13.3	61.9 ± 11.3	64.6 ± 12.2
Triglyceride (mg/dL)	49.8 ± 15.2	61.6 ± 26.4	47.1 ± 22.9	61.0 ± 28.5
Calcium (mg/dL)	12.3 ± 0.7	13.4 ± 0.3 *	13.1 ± 0.4 *	13.6 ± 0.6 *
Phosphorus (mg/dL)	10.7 ± 0.8	10.3 ± 0.7	10.8 ± 0.9	11.7 ± 1.8
Sodium (meq/L)	145.5 ± 1.0	146.2 ± 1.2	146.1 ± 1.4	144.3 ± 3.0
Potassium (meq/L)	7.1 ± 0.9	6.7 ± 0.7	7.3 ± 1.3	8.0 ± 2.3
Chloride (meq/L)	102.6 ± 1.3	101.0 ± 1.4 *	101.9 ± 1.7	100.0 ± 0.9 *
Globulin (g/dL)	2.1 ± 0.2	2.2 ± 0.2	2.1 ± 0.2	2.2 ± 0.2
Total bilirubin (mg/dL)	<0.04	<0.04	<0.04	<0.04
Female				
Glucose (mg/dL)	206.6 ± 52.6	206.8 ± 44.5	183.5 ± 55.4	203.0 ± 71.6
BUN (mg/dL)	15.3 ± 1.5	15.0 ± 2.1	15.4 ± 1.2	16.4 ± 1.7
Creatinine (mg/dL)	0.83 ± 0.05	0.77 ± 0.05 *	0.76 ± 0.05 *	0.77 ± 0.05 *
AST (U/L)	108.2 ± 38.0	130.4 ± 91.6	97.8 ± 26.5	116.1 ± 40.9
ALT (U/L)	36.9 ± 11.1	36.2 ± 15.2	37.2 ± 20.2	38.7 ± 12.7
Total protein (g/dL)	7.9 ± 0.4	7.8 ± 0.6	7.6 ± 0.5	7.8 ± 0.5
Albumin (g/dL)	6.0 ± 0.4	5.8 ± 0.5	5.7 ± 0.6	5.9 ± 0.5
ALP (U/L)	30.6 ± 7.8	32.7 ± 5.1	36.0 ± 11.2	29.2 ± 4.3
γ-GT (U/L)	<2.0	<2.0	<2.0	<2.0
Cholesterol (mg/dL)	80.0 ± 19.7	81.1 ± 9.0	70.2 ± 13.4	78.6 ± 15.2
Triglyceride (mg/dL)	43.2 ± 13.8	49.7 ± 23.0	32.0 ± 15.7	39.8 ± 18.7
Calcium (mg/dL)	13.4 ± 0.6	14.0 ± 0.5 *	13.7 ± 0.3	14.2 ± 0.6 *
Phosphorus (mg/dL)	9.9 ± 0.8	10.3 ± 1.4	10.0 ± 1.2	10.3 ± 1.1
Sodium (meq/L)	145.0 ± 0.9	145.7 ± 1.3	145.6 ± 1.5	144.9 ± 1.4
Potassium (meq/L)	9.2 ± 1.3	8.7 ± 2.0	9.3 ± 1.7	9.0 ± 1.3
Chloride (meq/L)	103.8 ± 1.5	103.3 ± 1.5	102.9 ± 1.7	101.8 ± 1.5 *
Globulin (g/dL)	2.0 ± 0.2	2.0 ± 0.2	1.9 ± 0.3	1.8 ± 0.2
Total bilirubin (mg/dL)	<0.04	<0.04	<0.04	<0.04

BUN: blood urea nitrogen. ^a^ Data expressed as mean ± S.D., *n* = 10. * Significant difference from control group (*p* < 0.05).

**Table 10:** Absolute organ weights (g) of male and female rats treated with NTU 101 powder via gastric gavage for 90 consecutive days ^a^.

	Dose			
Item (g)	Control	Low Dose (500 mg NTU 101/kg)	Medium Dose (1000 mg NTU 101/kg)	High Dose (2000 mg NTU 101/kg)
Male				
Body weight (before)	218.0 ± 8.7	218.0 ± 8.0	217.8 ± 7.6	217.7 ± 7.7
Body weight (after) ^b^	538.1 ± 49.8	543.9 ± 27.1	535.1 ± 39.3	519.6 ± 41.5
Testis	3.478 ± 0.316	3.454 ± 0.289	3.474 ± 0.417	3.550 ± 0.282
Adrenal gland	0.057 ± 0.009	0.060 ± 0.010	0.060 ± 0.007	0.058 ± 0.006
Spleen	0.739 ± 0.107	0.807 ± 0.153	0.873 ± 0.130 *	0.845 ± 0.120
Kidney	3.7171 ± 0.351	4.072 ± 0.715	4.032 ± 0.366	4.070 ± 0.525
Heart	1.693 ± 0.175	1.780 ± 0.170	1.677 ± 0.118	1.743 ± 0.133
Brain	2.101 ± 0.080	2.182 ± 0.088	2.147 ± 0.122	2.224 ± 0.068 *
Liver	14.475 ± 1.696	15.360 ± 2.948	14.978 ± 1.360	15.416 ± 2.054
Female				
Body weight (before)	172.8 ± 9.2	172.8 ± 9.2	172.7 ± 8.7	172.7 ± 8.7
Body weight (after) ^b^	253.2 ± 12.5	263.3 ± 22.6	266.2 ± 19.9	253.3 ± 16.9
Ovary	0.094 ± 0.025	0.087 ± 0.016	0.083 ± 0.020	0.087 ± 0.017
Adrenal gland	0.068 ± 0.018	0.066 ± 0.015	0.063 ± 0.015	0.066 ± 0.009
Spleen	0.492 ± 0.057	0.537 ± 0.125	0.511 ± 0.085	0.499 ± 0.087
Kidney	2.228 ± 0.145	2.294 ± 0.379	2.170 ± 0.139	2.214 ± 0.287
Heart	1.039 ± 0.080	1.043 ± 0.065	0.992 ± 0.093	1.004 ± 0.117
Brain	1.998 ± 0.074	2.140 ± 0.353	1.991 ± 0.059	1.986 ± 0.070
Liver	8.963 ± 0.751	8.673 ± 0.782	7.992 ± 0.456 *	8.442 ± 0.960

^a^ Data expressed as mean ± S.D., *n* = 10. ^b^ Body weight of the rats after overnight fasting at the end of the study. * Significant difference from control group (*p* < 0.05). Range of normal reference: spleen = 0.065~0.91 (g), brain = 1.97~2.41 (g), Super Laboratory historical values.

## Data Availability

All data included in this study are available upon request by contacting the corresponding authors.

## Supplementary Materials

The following supporting information can be downloaded at https://www.mdpi.com/article/10.3390/cimb46110734/s1: Table S1. API 50 CHL panel results of *Lacticaseibacillus paracasei* subsp. *paracasei* NTU 101; Table S2. Whole-genome sequence information of NTU 101, including basic data and quality evaluation of genome assembly, was compared with that of *Lacticaseibacillus paracasei* subsp. *paracasei* JCM 8130^T^; Figure S1. *Lacticaseibacillus paracasei* subsp. *paracasei* NTU 101 under (A) light microscope, (B) scanning electron microscope; Figure S2. COG functional classification of NTU 101; Figure S3. The phylogenetic tree of NTU 101 and its closely related type strains within the genus *Lacticaseibacillus*, constructed based on the 16S rDNA sequences of each strain; Figure S4. The phylogenomic tree of NTU 101 and its closely related type strains within the genus *Lacticaseibacillus*, constructed based on the whole-genome sequences of each strain.
