# Supplementary material for: Comprehensive Safety Assessment of Lacticaseibacillus paracasei subsp. paracasei NTU 101 Through Integrated Genotypic and Phenotypic Analysis
Source: Curr Issues Mol Biol. 2024 Nov 1;46(11):12354–74. doi: 10.3390/cimb46110734 (PMC11593238; doi:10.3390/cimb46110734)
Supplement: Supplementary file 1 [file cimb-46-00734-s001.zip › cimb-3252288-supplementary.pdf]

Table S1. API 50 CHL panel results of *Lacticaseibacillus paracasei* subsp. *paracasei* NTU 101.

Tests	Ingredients	Results	Tests	Ingredients	Results
	Control	−	ESC	Esculin, ferric citrate	+
GLY	Glycerol	−	SAL	Salicin	+
ERY	Erythritol	−	CEL	D-Cellobiose	+
DARA	D-Arabinose	−	MAL	D-Maltose	+
LARA	L-Arabinose	−	LAC	D-Lactose (Bovine origin)	+
RIB	D-Ribose	+	MEL	D-Melibiose	−
DXYL	D-Xylose	−	SAC	D-Saccharose (Sucrose)	+
LXYL	L-Xylose	−	TRE	D-Trehalose	+
ADO	D-Adonitol	+	INU	Inulin	−
MDX	Methyl-βD-xylopyranoside	−	MLZ	D-Melezitose	+
GAL	D-Galactose	+	RAF	D-Raffinose	−
GLU	D-Glucose	+	AMD	Amidon (Starch)	−
FRU	D-Fructose	+	GLYG	Glycogen	−
MNE	D-Mannose	+	XLT	Xylitol	−
SBE	L-Sorbose	+	GEN	Gentiobiose	+
RHA	L-Rhamnose	−	TUR	D-Turanose	+
DUL	Dulcitol	−	LYX	D-Lyxose	−
INO	Inositol	−	TAG	D-Tagatose	+
MAN	D-Mannitol	+	DFUC	D-Fucose	−
SOR	D-Sorbitol	+	LFUC	L-Fucose	−
MDM	Methyl-αD-mannopyranoside	−	DARL	D-Arabitol	−
MDG	Methyl-αD-glucopyranoside	−	LARL	L-Arabitol	−
NAG	N-Acetyl-glucosamine	+	GNT	Potassium Gluconate	+
AMY	Amygdalin	+	2KG	Potassium 2-Ketogluconate	−
ARB	Arbutin	+	5KG	Potassium 5-Ketogluconate	−

+: Positive reaction; -: Negative reaction; /: Borderline.

Table S2. Whole-genome sequence information of NTU 101, including basic data and quality evaluation of genome assembly, was compare with that of *Lacticaseibacillus paracasei* subsp. *paracasei* JCM 8130^T.

Strain	NTU 101	JCM 8130^T
Basic information		
Accession No.	GCA_002901165.3	GCA_000829035.1
Genome size (bp)	3,061,587	3,017,804
GC content (%)	46.5	46.5
No. contigs	2	3
Plasmid number	1	2
Genes	2956	2,976
CDS	2879	2897
tRNA	59	61
rRNA	15	15
Assembly quality		
CheckM		
Completeness	91.88%	96.27%
Contamination	4.13%	4.74%

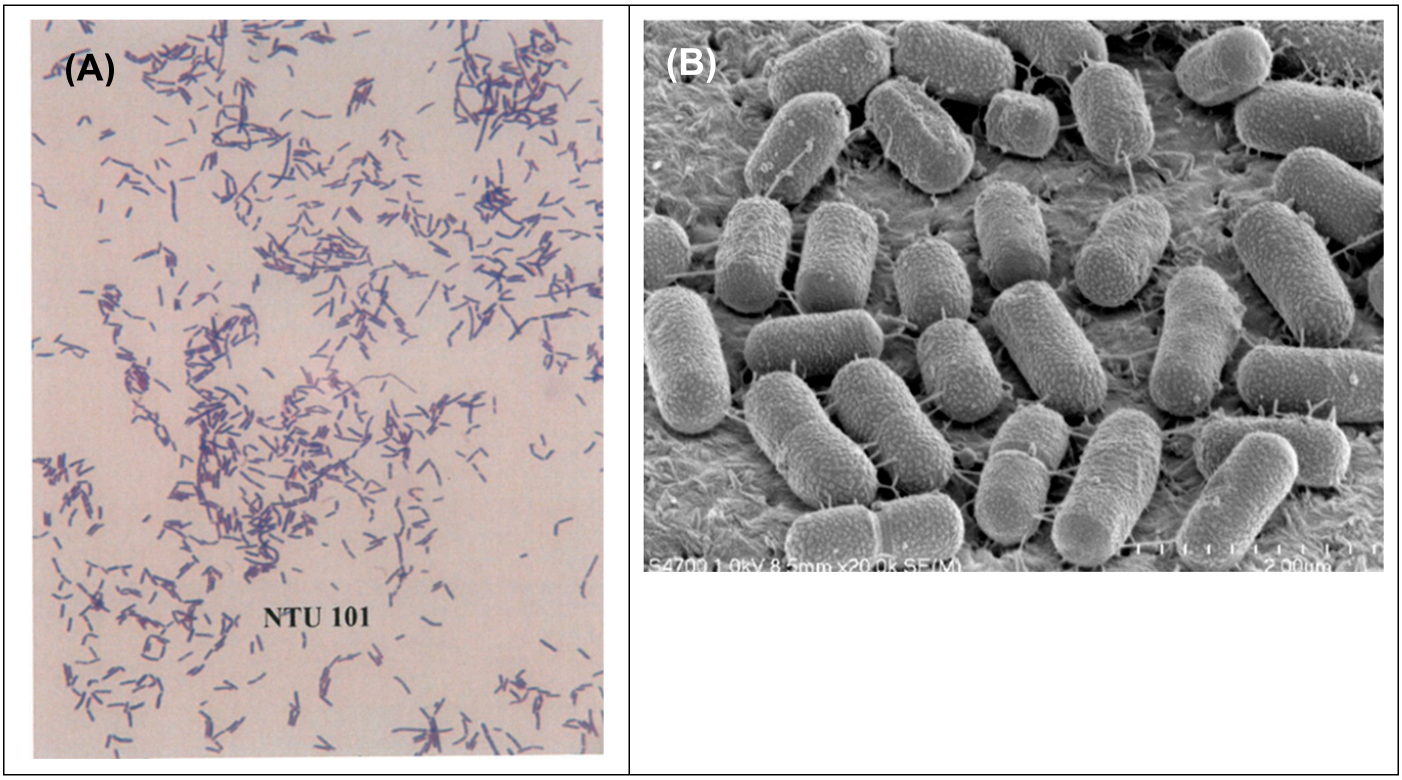

Figure S1. *Lacticaseibacillus paracasei* subsp. *paracasei* NTU 101 under (A) light microscope. (B) scanning electron microscope.

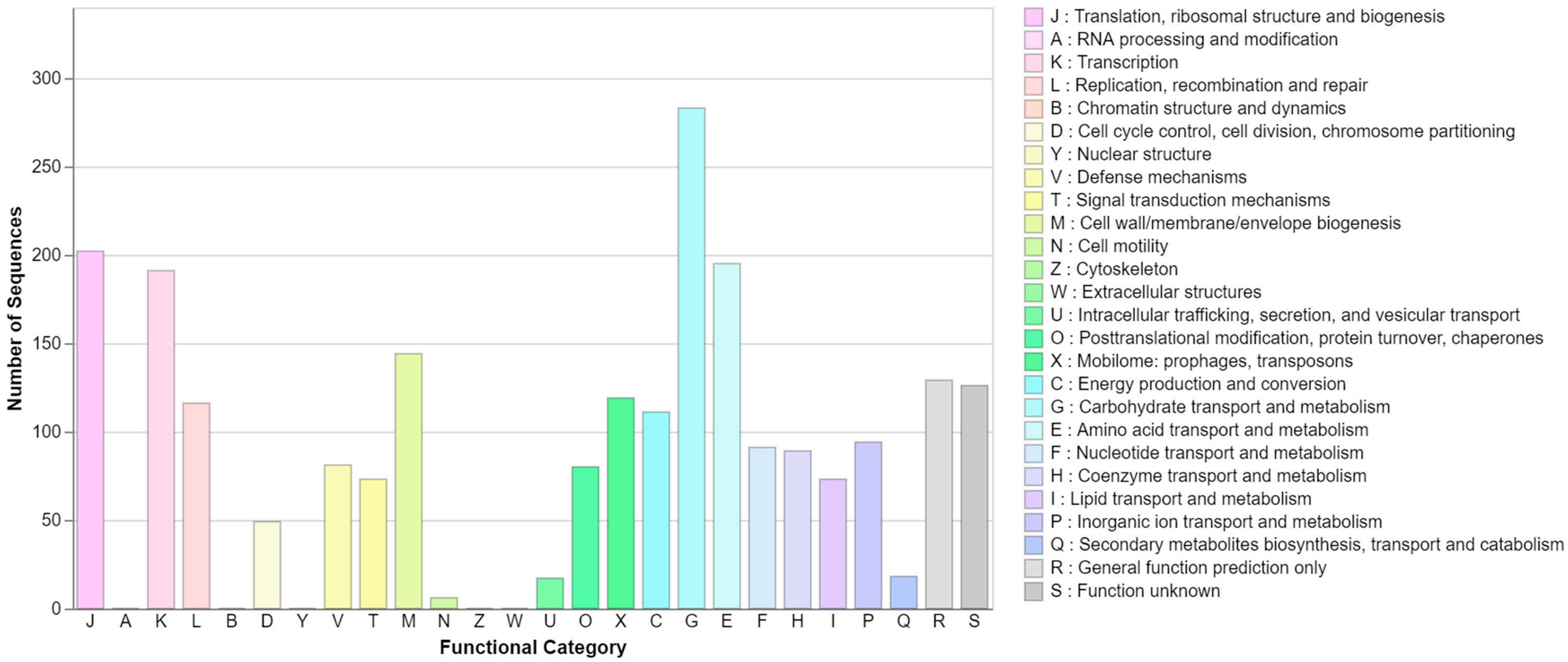

Figure S2. COG functional classification of NTU 101.

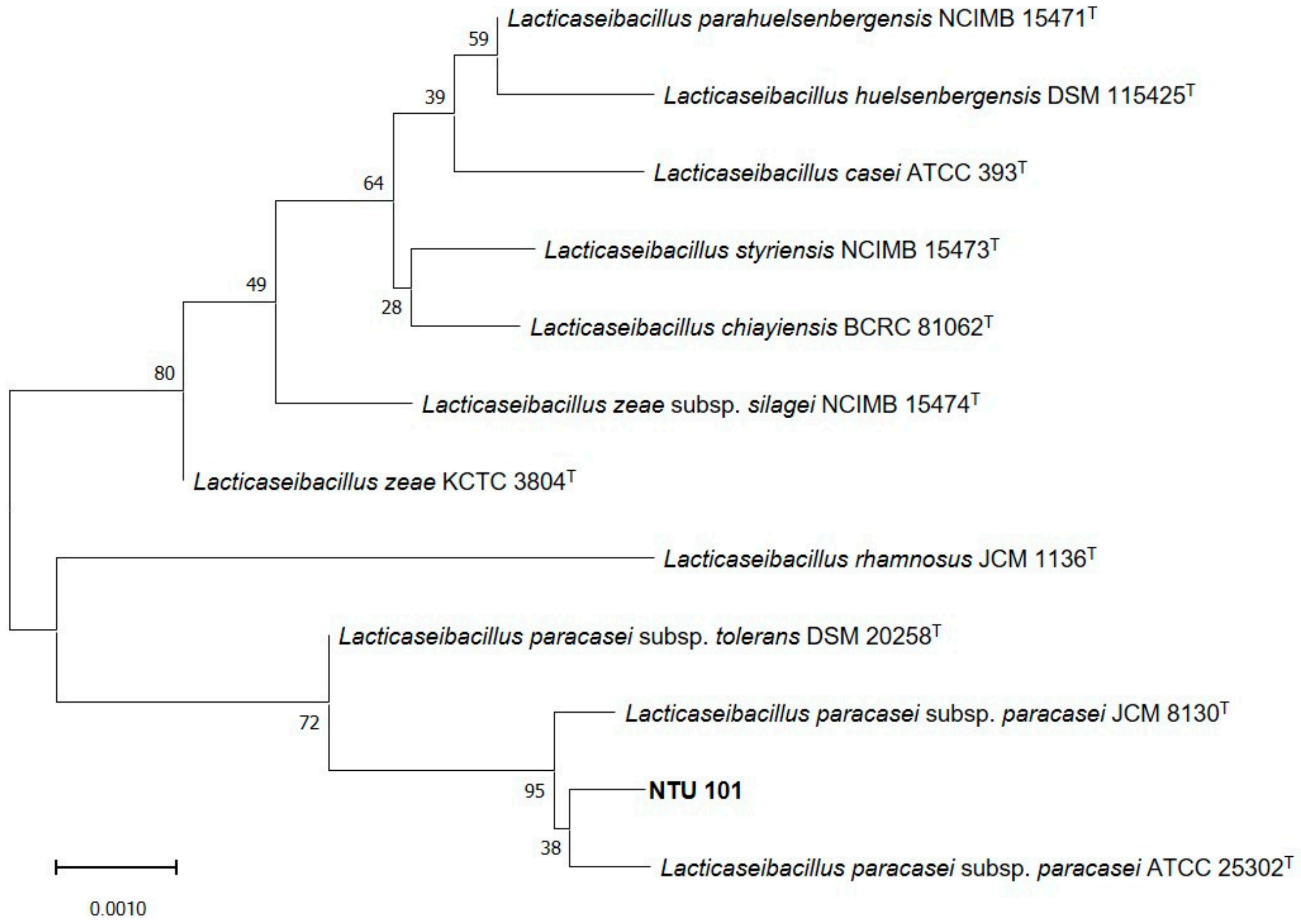

Figure S3. The phylogenetic tree of NTU 101 and its closely related type strains within the genus *Lacticaseibacillus*, constructed based on the 16S rDNA sequences of each strain. According to the TYGS report, tree inferred with FastME 2.1.6.1 from GBDP distances calculated from 16S rDNA sequences. The branch lengths are scaled in terms of GBDP distance formula *d5*. The numbers above branches are GBDP pseudo-bootstrap support values > 60 % from 100 replications, with an average branch support of 58.2 %. The tree was rooted at the midpoint.

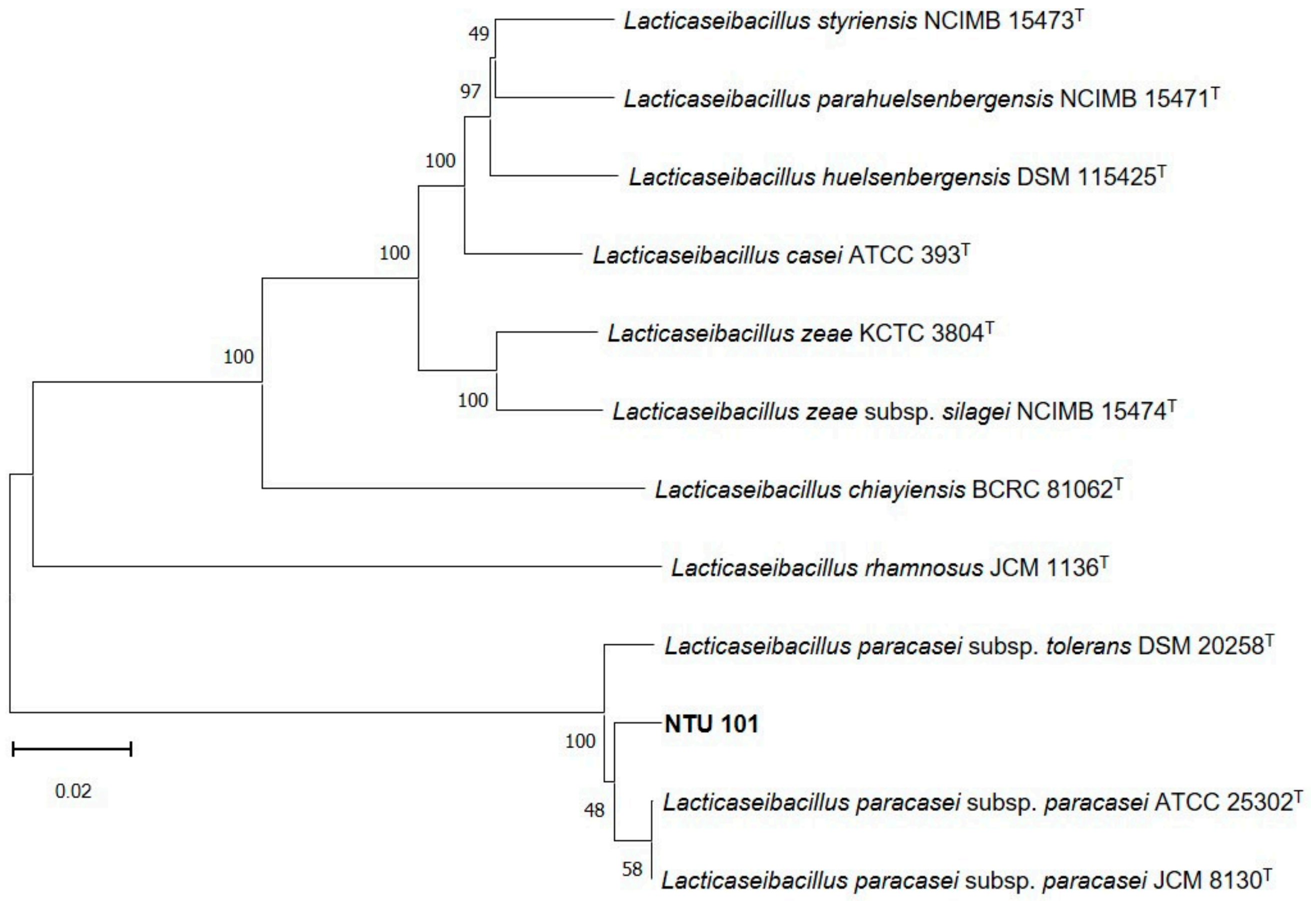

Figure S4. The phylogenomic tree of NTU 101 and its closely related type strains within the genus *Lacticaseibacillus*, constructed based on the whole-genome sequences of each strain. Using the same method as for the calculation of the 16S rDNA phylogenetic tree, an average branch support of 83.6% was obtained. The tree was rooted at the midpoint.
